# LAG‐3 transcriptomic expression patterns across malignancies: Implications for precision immunotherapeutics

**DOI:** 10.1002/cam4.6000

**Published:** 2023-05-03

**Authors:** Jacob J. Adashek, Shumei Kato, Daisuke Nishizaki, Hirotaka Miyashita, Pradip De, Suzanna Lee, Sarabjot Pabla, Mary Nesline, Jeffrey M. Conroy, Paul DePietro, Scott Lippman, Razelle Kurzrock

**Affiliations:** ^1^ Department of Oncology The Sidney Kimmel Comprehensive Cancer Center The Johns Hopkins Hospital Baltimore Maryland USA; ^2^ Center for Personalized Cancer Therapy and Division of Hematology and Oncology, Department of Medicine UC San Diego Moores Cancer Center La Jolla California USA; ^3^ Dartmouth Cancer Center, Hematology and Medical Oncology Lebanon New Hampshire USA; ^4^ Avera Cancer Institute Sioux Falls South Dakota USA; ^5^ OmniSeq Inc. Buffalo New York USA; ^6^ WIN Consortium San Diego California USA; ^7^ Department of Oncology MCW Cancer Center Milwaukee Wisconsin USA; ^8^ Department of Oncology University of Nebraska Omaha Nebraska USA

**Keywords:** biomarkers, clinical trials, experimental therapeutics, immune checkpoints, immunology

## Abstract

**Background:**

Lymphocyte activation gene 3 (LAG‐3) or CD223 is a transmembrane protein that serves as an immune checkpoint which attenuates T‐cell activation. Many clinical trials of LAG‐3 inhibitors have had modest effects, but recent data indicate that the LAG‐3 antibody relatlimab, together with nivolumab (anti‐PD‐1), provided greater benefit than nivolumab alone in patients with melanoma.

**Methods:**

In this study, the RNA expression levels of 397 genes were assessed in 514 diverse cancers at a clinical‐grade laboratory (OmniSeq: https://www.omniseq.com/). Transcript abundance was normalized to internal housekeeping gene profiles and ranked (0–100 percentile) using a reference population (735 tumors; 35 histologies).

**Results:**

A total of 116 of 514 tumors (22.6%) had high LAG‐3 transcript expression (≥75 percentile rank). Cancers with the greatest proportion of high LAG‐3 transcripts were neuroendocrine (47% of patients) and uterine (42%); colorectal had among the lowest proportion of high LAG‐3 expression (15% of patients) (all *p* < 0.05 multivariate); 50% of melanomas were high LAG‐3 expressors. There was significant independent association between high LAG‐3 expression and high expression of other checkpoints, including programmed death‐ligand 1 (PD‐L1), PD‐1, and CTLA‐4, as well as high tumor mutational burden (TMB) ≥10 mutations/megabase, a marker for immunotherapy response (all *p* < 0.05 multivariate). However, within all tumor types, there was inter‐patient variability in LAG‐3 expression level.

**Conclusions:**

Prospective studies are therefore needed to determine if high levels of the LAG‐3 checkpoint are responsible for resistance to anti‐PD‐1/PD‐L1 or anti‐CTLA‐4 antibodies. Furthermore, a precision/personalized immunotherapy approach may require interrogating individual tumor immunograms to match patients to the right combination of immunotherapeutic agents for their malignancy.

## Graphical abstract

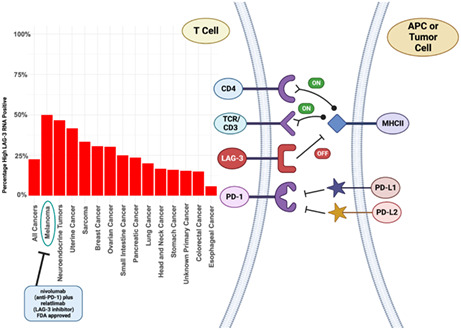

## 1 INTRODUCTION

Lymphocyte activation gene 3 (LAG‐3) or CD223 is an immune checkpoint found on various immune modulating cells: regulatory T cells (Tregs), natural killer cells, plasmacytoid dendritic cells, and CD4^+^, CD8^+^ T cells.^1^ LAG‐3 is a Type I transmembrane protein with structural similarities to CD4. LAG‐3 functions as an inhibitory co‐receptor and is important in autoimmunity as well as immunity related to infections and cancer.^2^ To avert tissue damage due to immune responses against self, immune cells are under strict check by multiple mechanisms, including inhibitory co‐receptors (checkpoints) such as PD‐1, CTLA‐4, and LAG‐3. Cancer cells hijack these checkpoints, inactivating the immune response, and permitting the cancer to survive and thrive. Not surprisingly, PD‐1/PD‐L1, CTLA‐4, and LAG‐3 checkpoints are therefore therapeutic targets, and antibodies that inhibit them (immune checkpoint inhibitors) have shown activity in a variety of cancers and are now approved.^3^, ^4^, ^5^

**FIGURE 1:**
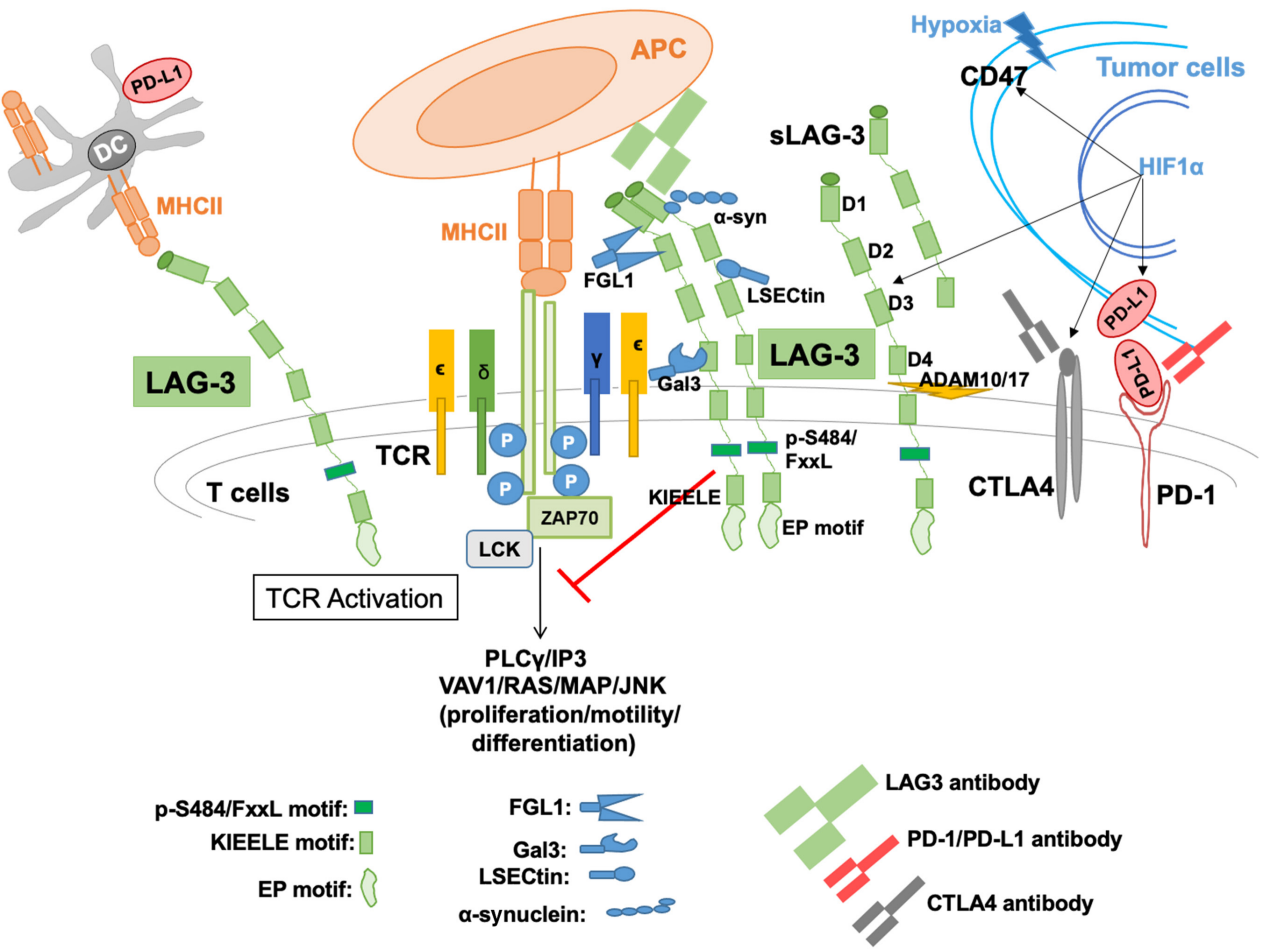
LAG‐3 (CD223) interaction with immune system and cancer progression. Lymphocyte‐associated gene 3 (LAG‐3) is a 70 kDa transmembrane glycoprotein, acts as co‐receptor found on activated T‐ cells, NK‐cells and also on B‐cells. LAG‐3 is comprised of four extracellular domains, D1, D2, D3, and D4, with D4 located closest to the cell membrane, and D1 being most distal. LAG‐3 contains specific binding sites (D1 and may also D2) for high affinity of MHCII, and functions as an inhibitor for T‐cell signaling/activation. It also binds with FGL‐1, α‐synuclein fibrils (α‐syn), the lectins galectin‐3 (Gal‐3), and lymph node sinusoidal endothelial cell C‐type lectin (LSECtin). It has been reported that FGL1 binds to LAG‐3 at D1 and D2, while Gal3 as well as LSECtin bind to N‐linked glycans at glycosylation sites, and α‐syn has been shown to bind to the DI domain.^8^ The cytoplasmic tail of LAG‐3 is indispensable to LAG‐3‐medited inhibition of T‐cells signaling/activation. Interestingly, LAG‐3 does not encode any of the classical inhibitory motif generally found in other immune‐modulatory receptors for example, immune‐receptor tyrosine‐based inhibitory motifs. However, at the membrane proximal region, it consists FxxL motif which plays the greater inhibitory role.^9^ The cytoplasmic domain contains three characteristic features first highlighted through conservation between human and mouse LAG‐3: (1) serine phosphorylation motif (S454), (2) EP motif (glutamic acid‐proline dipeptide), and (3) the lysine residue containing KIEELE motif may be essential for LAG‐3‐mediated inhibition. Tumor hypoxia‐mediated HIF1α stabilization upregulates the expression of LAG‐3, CTLA4, and also PD‐L1.^10^ Soluble LAG‐3 (sLAG‐3) is produced when membrane‐bound LAG‐3 is cleaved by matrix metalloproteinase ADAM10 and/or ADAM17 between D4 and the transmembrane domain.

LAG‐3 acts as an immune modulatory molecule via multiple mechanisms. In carcinogenesis, the expression of the LAG‐3 molecule increases T‐cell exhaustion, increasing the immune suppressive cytokine release thus leading to decreased tumor killing.^6^ The LAG‐3 cell surface receptor binds to the major histocompatibility complex (MHC) class II with higher affinity toward CD4 and thus results in T‐cell deactivation.^7^ LAG‐3 expression on tumor‐infiltrating lymphocytes and Tregs contributes to tumor immune evasion by dampening immune killing ability intratumorally, as well as by leading to increased expression of immune suppressive cytokines IL‐10 and TGF‐beta, causing further immune suppression and tumor escape from immune surveillance (Figure 1).^8^, ^9^, ^10^, ^11^ Importantly, LAG‐3 synergizes with PD‐1 to regulate T‐cell function in order to abet tumoral immune escape.^12^ There is also evidence that LAG‐3 expression is correlated with tumor mutational burden (TMB); in microsatellite instability‐high (MSI‐H) tumors, there is upregulation of LAG‐3.^13^ Both high TMB and MSI‐H status have been associated with responsiveness to anti‐PD‐1/PD‐L1 checkpoint inhibitors.^14^, ^15^, ^16^ Expression of LAG‐3 is also differentially upregulated, regardless of tumor histology, when alterations are present in *CDKN2A*, *EZH2*, and *MPL* genes.^17^

There have been multiple interventional clinical trials employing LAG‐3 inhibitors (Table 1).^3^, ^18^, ^19^, ^20^, ^21^, ^22^, ^23^, ^24^ However, trials including LAG‐3‐blocking drugs seem to have modest effects on outcomes.^1^ Even so, recent data in patients with metastatic melanoma revealed that the LAG‐3 blocking antibody relatlimab, combined with nivolumab (anti‐PD‐1), prolonged progression‐free survival more than nivolumab alone (10.1 vs. 4.6 months; hazard ratio 0.75; 95% confidence interval [CI] 0.62–0.92; *p* = 0.006).^3^ These results led to Food and Drug Administration (FDA) approval of nivolumab together with relatlimab for unresectable or metastatic melanoma in March 2022 (https://www.fda.gov/drugs/resources‐information‐approved‐drugs/fda‐approves‐opdualag‐unresectable‐or‐metastatic‐melanoma).

**TABLE 1:** Examples of clinical trials evaluating LAG‐3 inhibitors.

Drug name/company mechanism	Combination strategy	Phase	Cancer type	Results	Biomarker selection (yes/no [type of biomarker])	Comment (status as of June 7, 2022)	Reference/NCT number
*LAG‐3 antibodies*							
Relatlimab/Bristol‐Myers Squibb	Nivolumab	II–III	Melanoma	PFS 10.1 versus 4.6 months for nivolumab alone (HR 0.75; 95% CI, 0.62–0.92; *p* = 0.006)	No	Completed	3/NCT03470922
LAG‐3 antibody						Relatlimab/nivolumab combination approved by FDA for melanoma in March 2022	
Relatlimab	Nivolumab	I	Melanoma	ORR 16% [5/31]	No	Active, not recruiting	3/NCT01968109
Relatlimab	Nivolumab (anti‐PD‐1)	II	Mismatch repair deficient solid tumors resistant to prior anti‐PD‐L1 therapy	Not reported	No	Recruiting	NCT03607890
Relatlimab	Nivolumab (anti‐PD‐1)	II	Chordoma	Not reported	No	Recruiting	NCT03623854
Relatlimab	Nivolumab (anti‐PD‐1)	II	Microsatellite‐stable advanced colorectal cancer	Not reported	No	Recruiting	NCT03642067
Relatlimab	Nivolumab (anti‐PD‐1)	II	Uveal melanoma	Not reported	No	Recruiting	NCT04552223
Relatlimab	Nivolumab (anti‐PD‐1)	I	Hepatocellular carcinoma	Not reported	No	Recruiting	NCT04658147
Relatlimab	Nivolumab (anti‐PD‐1)	I	Esophageal or gastric cancer	Not reported	No	Recruiting	NCT03610711
Relatlimab	Ipilimumab (anti‐CTLA‐4)	I	Melanoma	Not reported	No	Recruiting	NCT03978611
Sym022/Symphogen antibody (anti‐LAG‐3 that blocks interaction with MHDII)	None	I	Advanced solid tumors or lymphomas	Not reported	No	Completed	NCT03489369
GSK2831781/ GlaxoSmithKline LAG‐3 antibody	None	N/a	Healthy volunteers	N/a	No	Active, not recruiting	NCT03965533
Favezelimab/Merck LAG‐3 antibody	Pembrolizumab (anti‐PD‐1)	I	Microsatellite stable colorectal cancer	ORR: 6.3% DOR: 10.6 months [5/80]	No	Active, not recruiting	18/NCT02720068
LAG525/Novartis Oncology humanized LAG‐3 antibody	Spartalizumab (anti‐PD‐1)	II	Advanced cancers	CBR24 in NET: 86% [6/7] SCLC: 27% [4/15] DLBCL: 43% [3/7]	No	Completed	19/NCT03365791
REGN3767/ Regeneron Pharmaceuticals, Inc. LAG‐3 antibody	Cemiplimab (anti‐PD‐1)	I	Advanced cancers	Not reported	No	Recruiting	NCT03005782
*Eftilagimod alpha* *Soluble LAG‐3 fusion protein*							
Eftilagimod alpha/Immutep Soluble LAG‐3 fusion protein	None	I	Renal cell carcinoma	No responses reported	No	Completed	20/NCT00351949
Eftilagimod alpha	Paclitaxel	I/II	Breast	ORR 50% [15/30]	No	Completed	21/NCT00349934
Eftilagimod alpha	MART‐1 analog peptide vaccinations	I	Melanoma	Increase in MART‐1 CD8^+^ T cells 83% of patients ORR: 0% [0/6]	No	Completed	22/NCT00324623
Eftilagimod alpha	Pembrolizumab (anti‐PD‐1)	I	Melanoma	ORR anti‐PD‐1 refractory: 33% [6/18] Median PFS anti‐PD‐1 refractory: 4.7 months ORR, anti‐PD‐1 naïve: 50% [3/6] Median PFS: Not reached	No	Completed	23/NCT02676869
Eftilagimod alpha	Pembrolizumab (anti‐PD‐1)	II	Non‐small cell lung cancer	ORR by blinded independent central review: 42% (95% CI 25.5–59.2) Median PFS: 8.2 months Median OS not yet reached	No	Active, not recruiting	24/NCT03625323
Eftilagimod alpha/	Montanide (adjuvant)	I	HLA‐A2 positive Stage II–IV melanoma	Terminated due to low enrollment rate	No	Terminated due to low enrollment rate	NCT01308294

Abbreviations: ADCC, antibody‐dependent cellular cytotoxicity; CBR24, clinical benefit rate at 24 weeks; CDC, complement‐dependent cytotoxicity; DCR, disease control rate (complete response + partial response + stable disease >12 weeks); DLBCL, diffuse large B‐cell lymphoma; DOR, duration of response; HR, hazard ratio for progression or death; MHC, major histocompatibility complex class II; MOA, mechanism of action; N/a, not applicable; NET, neuroendocrine tumor; NR, not reached; ORR, objective response rate; PFS, progression‐free survival; SCLC, small cell lung cancer.

Herein we examine the landscape of the LAG‐3 transcriptomic profile in 514 patients with cancer, and potential therapeutic implications of the heterogenous portfolios observed.

## 2 METHODS

### 2.1 Patients

The RNA expression levels of LAG‐3 from 514 solid tumor samples (Table S1) from the University of California San Diego (UCSD) Moores Center for Personalized Cancer Therapy clinic were analyzed at a Clinical Laboratory Improvement Amendments (CLIA)‐licensed and College of American Pathologist (CAP)‐accredited clinical laboratory–OmniSeq (https://www.omniseq.com/). The NGS and transcriptome panel used in OmniSeq analyses are commercially available through Thermo Fisher and includes immune response relevant genes. This assay allows for quantitative evaluation of the expression of different antigen presentation, checkpoint pathways, leukocyte subsets, and tumor progression.^25^ Data collection included histological types of primary cancer, patients' age, sex, TMB, and programmed death‐ligand 1 (PD‐L1) status. If multiple unique samples were analyzed from the same patient different days, the earlier timestamped sample was included in this analysis. This study was part of a clinical grade assay and included any patient with advanced cancer for whom the physician ordered immunomic analysis.

### 2.2 Sampling of tissue and analysis of cancer immunity markers

The samples were provided after tumor collection (formalin‐fixed, paraffin‐embedded [FFPE]), and evaluated by RNA sequence at OmniSeq laboratory. The RNA was extracted from FFPE using truXTRAC FFPE extraction kit (Covaris, Inc.), mostly following the manufacturer's instructions. After purification, the RNA was dissolved in 50 μL water and the yield was measured via Quant‐iT RNA HS assay (Thermo Fisher Scientific), per the manufacturer's recommendation. For library preparation, the predefined titer of 10 ng of RNA was deemed acceptable. Torrent Suite's plugin immuneResponseRNA (v5.2.0.0) 34 was employed for RNA expression absolute read count estimation. Background subtraction, normalization, and percentile ranking was performed using custom scripts.^25^

Transcript abundance was normalized to an internal housekeeping gene profile dataset and ranked (0–100 percentile) in a standardized manner to a reference dataset of 735 tumors spanning 35 tumor histologies. The expression profiles were stratified by transcript abundance rank values into “low” (0–74) and “high” (75–100) percentile; or low defined as 0–24 percentile, moderate defined as 25–74, and high defined as 75–100 percentile LAG‐3 RNA expression rank.

### 2.3 Definition of variables

To analyze TMB, genomic DNA was obtained from qualified FFPE tumors (>30% tumor nuclei) by means of the truXTRAC FFPE extraction kit (Covaris) with 10 ng DNA input for library preparation. DNA Libraries were readied with Ion AmpliSeq targeted sequencing chemistry employing the Comprehensive Cancer Panel, followed by enrichment and template preparation utilizing the Ion Chef system, and sequencing on the Ion S5XL 540 chip (Thermo Fisher Scientific). After removal of synonymous variants, germline variants, indels and single nucleotide variants with <5% variant allele fraction, TMB is reported as eligible mutations per qualified panel size (mutations/megabase).

## 3 RESULTS

### 3.1 Population characteristics

**FIGURE 2:**
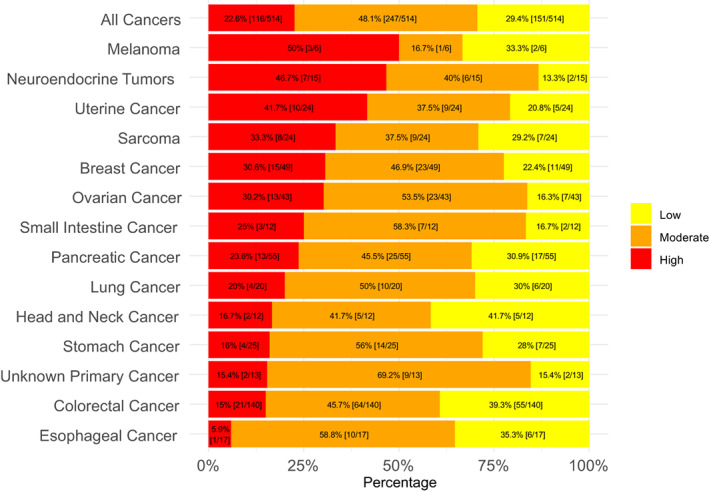
Expression of LAG‐3 among diverse cancers (*n* = 514). High defined as 75–100 percentile LAG‐3 RNA expression rank; moderate defined as 25–74; low defined as 0–24. Percentages in the bar graph are of patients with that designated level of LAG‐3 RNA expression. Transcript abundance was normalized to an internal housekeeping gene profile dataset and ranked (0–100 percentile rank) in a standardized manner to a reference dataset of 735 tumors spanning 35 tumor histologies. Tumor types with >10 samples were included (see Section 2). Although only a small number of melanomas were assessed, they were included in the figure because of the approval of the LAG‐3 inhibitor relatlimab for melanoma.

There were 514 tumors reflecting 31 different cancer types evaluated (Table S1). Their median age was 61; 310 (60%) were women. The most frequent tumor types assessed were colorectal cancer (*N* = 140 samples), pancreatic cancer (*N* = 55), breast cancer (*N* = 49), ovarian cancer (*N* = 43), lung cancer (*N* = 20), stomach cancer (*N* = 25), sarcoma (*N* = 24), uterine cancer (*N* = 24), and neuroendocrine cancer (*N* = 15). Figure 2 shows that LAG‐3 RNA expression differed between cancer types: LAG‐3 RNA expression was designated as “low” (0–24 percentile), “moderate” (25–74 percentile), and “high” (75–100 percentile). Among all samples (*N* = 514), 116 (22.6%) had high LAG‐3, 247 had moderate, and 151 had low expression. Neuroendocrine and uterine cancers most frequently had high LAG‐3 RNA expression (46.7% and 41.7% of tumors, respectively). Fifty percent of melanomas also had high LAG‐3 expression, with the caveat that only six melanomas were tested. Esophageal and colorectal cancer had the lowest frequency of high LAG‐3 expression (5.9% and 15% of tumors, respectively). Expression of LAG‐3 differed between tumors even within cancer types: Importantly, there was variability of LAG‐3 expression even within tumor types. For instance, while half of melanomas expressed high LAG‐3, 33.3% expressed low LAG‐3 (admittedly with small numbers of patients). Similarly, while 46.7% of neuroendocrine tumors expressed high LAG‐3, 13.3% expressed low LAG‐3, with the rest being moderate. This pattern reflecting individual variability was seen in all cancer types analyzed (Figure 2). High‐LAG‐3 RNA levels associated independently with uterine and neuroendocrine cancers, and with specific immunotherapy markers (increased TMB [>10 mutations/mb], high).

### 3.2 High‐LAG‐3 RNA levels associated independently with uterine and neuroendocrine cancers, and with specific immunotherapy markers (increased TMB [≥10 mutations/mb], high PD‐1, PD‐L1 and CTLA‐4 transcript expression)

The following variables were significantly associated with LAG‐3 RNA expression in univariate analysis: gender, PD‐L1, PD‐1, PD‐L2, CTLA‐4, TMB, colorectal cancer, uterine cancer, and neuroendocrine cancer (Table 2). Multivariate analysis was then performed on variables with *p* values ≤0.05 in univariate analysis to ascertain features independently correlated with LAG‐3 expression. All variables selected in univariate analysis except PD‐L2 remained associated with LAG‐3 expression in multivariate analysis.

**TABLE 2:** Univariate and multivariate analysis of LAG‐3 transcriptomic expression and clinical and immunomic features (*n* = 514 patients).

Feature^a^	*N* (%) of patients with “high” LAG‐3^b^	Odds ratio for “high” LAG‐3 (95% CI)	Univariate *p* value	Multivariate odds ratio for “high” LAG‐3 (95% CI)^c^	Multivariate *p* value
Age ≥ 61 years (*n* = 256) Age < 61 years (*n* = 258)	64 (25) 52 (20)	1.321 (0.872–2.001) 0.7573 (0.4999–1.1473)	0.1896		
Men (*N* = 203) Women (*N* = 310)	35 (17) 81 (26)	0.589 (0.3779–0.9179) 1.69 (1.08–2.6)	0.0194	0.50 (0.27–0.90) 2.0 (1.1–3.7)	*p* = 0.024 (“high” LAG‐3 less frequent in men)
“High” PD‐L1 (*n* = 67) “Low/Moderate” PD‐L1 (*n* = 447)	42 (63) 74 (17)	8.468 (4.9–14.7) 0.1181 (0.0678–0.2056)	<0.0001	4.31 (2.02–9.22) 0.232 (0.108–0.495)	*p* = 0.0002 (“high” LAG‐3 more frequent with “high” PD‐L1)
“High” PD‐1 (*n* = 93) “Low/Moderate” PD‐1 (*n* = 421)	58 (62) 58 (14)	10.4 (6.2730–17.1476) 0.0964 (0.0583–0.1594)	<0.0001	6.35 (3.13–13.1) 0.157 (0.076–0.319)	*p* < 0.0001 (“high” LAG‐3 more frequent with “high” PD‐1)
“High” PD‐L2 (*n* = 100) “Low/Moderate” PD‐L2 (*n* = 414)	46 (46) 70 (17)	4.19 (2.62–6.7) 0.2389 (0.1493–0.3821)	<0.0001	1.20 (0.56–2.47) 0.833 (0.405–1.786)	*p* = 0.628
“High” CTLA‐4 (*n* = 87) “Low/Moderate” CTLA‐4 (*n* = 427)	47 (54) 69 (16)	6.1 (3.72–9.99) 0.164 (0.1001–0.2689)	<0.0001	2.17 (1.02–4.52) 0.461 (0.221–0.98)	*p* = 0.041 (“high” LAG‐3 more frequent with “high” CTLA4)
TMB ≥10 mutations/mb (*n* = 33) TMB < 10 mutations/mb (*n* = 417)	14 (42) 83 (20)	2.97 (1.428–6.16) 0.3373 (0.1624–0.7005)	0.0036	5.33 (2.13–13.2) 0.188 (0.0758–0.469)	*p* = 0.0003 (“high” LAG‐3 more frequent with TMB ≥10)
Colorectal (*n* = 140) Other tumors (*n* = 374)	21 (15) 95 (25)	0.52 (0.308–0.871) 1.9295 (1.1482–3.2424)	0.0131	0.43 (0.20–0.87) 2.33 (1.15–5.0)	*p* = 0.023 (“high” LAG‐3 less frequent in colorectal cancer)
Breast cancer (*n* = 49) Other tumors (*n* = 465)	15 (31) 101 (22)	1.59 (0.833–3.03) 0.6289 (0.3296–1.2003)	0.160		
Ovarian cancer (*n* = 43) Other cancers (*n* = 471)	13 (30) 103 (22)	1.55 (0.779–3.08) 0.6459 (0.3251–1.2833)	0.2121		
Pancreatic cancer (*n* = 55) Other cancers (*n* = 459)	13 (24) 103 (22)	1.07 (0.553–2.07) 0.9347 (0.4833–1.8078)	0.8411		
Uterine cancer (*n* = 24) Other cancers (*n* = 490)	10 (42) 107 (22)	2.59 (1.09–5.95) 0.3861 (0.168–0.917)	0.026	3.28 (1.07–9.39) 0.305 (0.106–0.935)	*p* = 0.030 (“high” LAG‐3 more frequent in uterine cancer)
Sarcoma (*n* = 24) Other cancers (*n* = 490)	8 (33) 108 (22)	1.77 (0.737–4.243) 0.5654 (0.2357–1.3567)	0.202		
Neuroendocrine cancer (*n* = 15) Other cancers (n = 499)	7 (47) 109 (22)	3.131 (1.111–8.826) 0.3194 (0.1133–0.9004)	0.0309	3.98 (1.07–13.8) 0.351 (0.0725–0.935)	*p* = 0.032 (“high” LAG‐3 more frequent in neuroendocrine cancers)
Lung cancer (*n* = 20) Other cancers (*n* = 494)	4 (20) 112 (23)	0.853 (0.279–2.602) 1.1728 (0.3843–3.5791)	0.780		
Melanoma (*n* = 3) Other cancers (*n* = 511)	3 (50) 113 (22)	3.496 (0.639–19.111) 0.286 (0.0523–1.565)	0.1286		

*Note*: Microsatellite status was not tabulated because only 15 patients were microsatellite unstable. Tumor types with ≥40 samples and/or ≥23% of samples with high LAG‐3 (23% being the percent of all cancers with high LAG‐3) were assessed for odds ratio of high LAG‐3; lung cancer was also included because it is a prevalent tumor type in general population; melanoma was not included in the analysis because only six tumors were analyzed. Melanoma was included for LAG‐3 analysis in this table despite the small numbers of patients because of the recent FDA approval of the anti‐LAG‐3 relatlimab in melanoma^3^; ocular melanoma was not included in the six patients analyzed.

Abbreviations: mb, megabase; TMB, tumor mutational burden.

^a^ Total number of patients are less in some categories (e.g., TMB) because data was not available on all patients.

^b^ High LAG‐3 or PD‐L1 or PD‐1 or PD‐L2 or CTLA4 means ≥75 transcript expression percentile rank; “Low/Moderate” LAG‐3 or PD‐L1 or PD‐1 or PD‐L2 or CTLA‐4 means <75 percentile rank transcript expression.

^c^ Multivariate analysis was performed only among patients with available TMB (*n* = 450); variables with *p* value ≤0.05 were selected for multivariate analysis.

Regarding clinical characteristics, high levels of LAG‐3 were independently associated with female gender (but not age), and with uterine and neuroendocrine cancers; in contrast, colorectal cancer was significantly associated with less frequent high LAG‐3 levels.

Regarding biologic characteristics, high PD‐L1, high PD‐1, high CTLA‐4, and high TMB (≥ 10 mutations/mb) were significantly and independently correlated with high LAG‐3 transcripts. The strongest associations with high LAG‐3 were with high PD‐L1 (odds ratio; 95% CI univariate; *p* value multivariate) (odds ratio = 8.468; 95% CI 4.9–14.7; *p* = 0.0002) and high PD‐1 (odds ratio = 10.4; 95% CI 6.2730–17.1476; *p* < 0.0001).

## 4 DISCUSSION

In this analysis, high levels of LAG‐3 RNA expression were present in ~23% of 514 tumor samples. The observation that only a minority of tumors express LAG‐3 may explain why some trials of LAG‐3 antagonists report modest response rates (see Table 1). Furthermore, LAG‐3 expression was often accompanied by high PD‐1 and PD‐L1, as well as high CTLA‐4 expression, suggesting that dampening of checkpoint effect in many cancers might require combination therapy, that is, LAG‐3 inhibitors together with anti‐PD‐1/PD‐L1 agents and/or CTLA‐4 inhibitors. Indeed, many clinical trials with LAG‐3 inhibitors include an anti‐PD‐1 agent, and the LAG‐3 inhibitor relatlimab was approved by the FDA together with the anti‐PD‐1 agent nivolumab for melanoma based on a PFS of 10.1 versus 4.6 months for nivolumab alone (*p* = 0.006).^3^ In our study, 50% of melanomas expressed high LAG‐3 levels, consistent with the notion that LAG‐3 plays a role in shielding melanomas from immune reconnaissance.

In the current report, certain malignancies such as uterine and neuroendocrine cancers were more likely to express high LAG‐3 RNA levels in multivariate analysis (Table 2). Moreover, as mentioned, high LAG‐3 levels associated with high levels of PD‐1 and PD‐L1, both important in regulating cancer immunity. Of interest in this regard, a phase II study combining LAG525, a humanized anti‐LAG‐3 IgG4 antibody, with spartalizumab, an anti‐PD‐1 monoclonal antibody, reported the clinical benefit rate at 24 weeks in patients with advanced neuroendocrine tumors to be 86%; other tumor types also appeared to benefit, including advanced small cell lung cancer (clinical benefit rate at 24 weeks = 27%) and advanced diffuse‐large B cell lymphoma (clinical benefit rate at 24 weeks = 43%).^19^ Our analysis did not have sufficient samples from the latter two tumor types to evaluate LAG‐3 expression.

Our study highlights the importance of individualized therapies for patients, rather than population‐based approaches. Although certain tumor types and biologic characteristics were associated with high LAG‐3 in our analysis, there was high individual variability. For instance, while ~47% of neuroendocrine cancers expressed high LAG‐3, ~13% had low LAG‐3 levels (Figure 2). As another example, high LAG‐3 was significantly less frequent in colorectal cancer than in other tumor types (multivariate analysis), but still ~15% of colorectal tumors showed high LAG‐3. Prior studies have demonstrated that melanoma patients with a LAG‐3‐positive immune profile had poorer outcomes after immunotherapy (mostly anti‐PD‐1), with a median survival of 22.2 months compared to 75.8 months for those with the LAG‐negative immune profile (*p* = 0.031); these findings were validated in an independent cohort of patients with urothelial cancer.^26^ Similarly, our prior studies have shown that patients whose tumors were resistant to anti‐PD‐1/PD‐L1 agents more frequently expressed TIM‐3 and VISTA checkpoints.^27^ In essence, patients whose therapies were “missing the target” did not do well.^28^ Using an N‐of‐1 matching (drug to tumor target) approach has been successfully applied for genomic targets, offering enhanced efficacy^29^, ^30^ and may also inform how best to utilize immunotherapeutics.

The current work focused on the immune transcriptome. Precision medicine trials have already revealed that interrogating the transcriptome to inform clinical utility of drugs may also help improve outcomes.^29^ Although a good deal of precision oncology therapeutics has concentrated on genomics, the transcriptome is critically important for several reasons. First, some alterations (including amplifications) expressed at the genomic level are silenced at the RNA level, possibly leading to resistance.^31^ Indeed, ~13% of clinically relevant mutations found at the DNA level are silenced in RNA.^32^ Furthermore, fusions (which are often oncogenic drivers) can sometimes be better detected via RNA than DNA sequencing^33^ and the transcriptome can also be utilized to identify synthetic lethal interactions that can be exploited in the clinic therapeutic arena.^34^

The current study has several limitations. First, although 514 patients were analyzed, not all tumor types were available, and there were only a small number of melanomas. Second, there is a lack of clinical therapeutic correlates; in particular, future studies will need to examine the relationship between LAG‐3 and outcome after LAG‐3 inhibitors, which was not possible in this report since patients had not been treated with these agents. Even so, our investigation revealed novel associations between high LAG‐3 and biologic immune variables (high PD‐L1, PD‐1, CTLA‐4, and high TMB) and between LAG‐3 and clinical cancer types (with especially high levels in uterine and neuroendocrine cancers and low levels in colorectal cancer).

## 5 CONCLUSIONS

In conclusion, our study together with the existing literature suggests that identifying prosecutable biomarkers for immunotherapy is of paramount importance. However, the previously reported and ongoing LAG‐3 inhibitor trials do not generally employ biomarker selection for patient enrollment. To date, putative immune biomarkers include, but are not limited to, expression of PD‐L1, PD‐1, and TMB high, as well as certain MHC genotypes and T‐cell receptor repertoires.^14^, ^35^, ^36^, ^37^ In the case of LAG‐3, most malignancies do not have high LAG‐3 expression. However, high LAG‐3 levels are commonly found in neuroendocrine and uterine cancers, suggesting that these tumor types merit clinical trials with LAG‐3 inhibitors. Furthermore, high LAG‐3 RNA levels associate with high PD‐1/PD‐L1 and high CTLA‐4 levels, perhaps indicating that combinations of LAG‐3 inhibitors with antagonists of PD‐1/PD‐L1 and CTLA‐4 should be explored. The fact that high LAG‐3 expression often co‐exists with high (≥10 mutations/mb) TMB may imply that some of these cancers could be vulnerable to immune eradication in the presence of LAG‐3 combined with other cognate inhibitors. Most importantly, however, was the variability in LAG‐3 levels that we discerned across the cancer spectrum. In the future, using transcriptomics to identify the immunomic signature of individual tumors may be another step needed to fully develop the precision/personalized immunotherapy paradigm. Such a model would be analogous to the deployment of next generation sequencing to identify the genomic aberrations in individual cancers in order to pinpoint the optimal targeted therapy. Prospective trials that match patients with immunotherapies based on their tumor immunogram are warranted.

## AUTHOR CONTRIBUTIONS

**Jacob J. Adashek:** Conceptualization (equal); methodology (equal); visualization (equal); writing – original draft (equal); writing – review and editing (equal). **Shumei Kato:** Conceptualization (equal); writing – review and editing (equal). **Daisuke Nishizaki:** Data curation (equal); formal analysis (equal); writing – review and editing (equal). **Hirotaka Miyashita:** Data curation (equal); writing – review and editing (equal). **Pradip De:** Writing – review and editing (equal). **Suzanna Lee:** Data curation (equal); project administration (equal); writing – review and editing (equal). **Sarabjot Pabla:** Data curation (equal); writing – review and editing (equal). **Mary Nesline:** Writing – review and editing (equal). **Jeffrey M Conroy:** Writing – review and editing (equal). **Paul DePietro:** Writing – review and editing (equal). **Scott Lippman:** Writing – review and editing (equal). **Razelle Kurzrock:** Conceptualization (equal); supervision (equal); validation (equal); visualization (equal); writing – original draft (equal); writing – review and editing (equal).

## CONFLICT OF INTEREST STATEMENT

Jacob J. Adashek serves on the advisory board of CureMatch Inc. Shumei Kato serves as a consultant for Foundation Medicine. He receives speaker's fee from Roche and advisory board for Pfizer. He has research funding from ACT Genomics, Sysmex, Konica Minolta and OmniSeq. Hirotaka Miyashita and Daisuke Nishizaki have no conflicts of interest. Pradip De is a paid consultant of Viecure. Suzanna Lee has no conflicts of interest. Sarabjot Pabla, Mary Nesline, Jeffrey M. Conroy, Paul DePietro are employees of Omniseq. Scott M. Lippman is the co‐founder of io9 and is on Biological Dynamics, Inc. Scientific Advisory Board. Razelle Kurzrock has received research funding from Biological Dynamics, Boehringer Ingelheim, Debiopharm, Foundation Medicine, Genentech, Grifols, Guardant, Incyte, Konica Minolta, Medimmune, Merck Serono, Omniseq, Pfizer, Sequenom, Takeda, and TopAlliance; as well as consultant and/or speaker fees and/or advisory board for Actuate Therapeutics, AstraZeneca, Bicara Therapeutics, Biological Caris, Dynamics, Daiichi Sankyo, Inc., EISAI, EOM Pharmaceuticals, Iylon, Merck, NeoGenomics, Neomed, Pfizer, Prosperdtx, Roche, TD2/Volastra, Turning PointTherapeutics, X‐Biotech; has an equity interest in CureMatch Inc., CureMetrix, and IDbyDNA; serves on the Board of CureMatch and CureMetrix, and is a co‐founder of CureMatch.

## ETHICS STATEMENT AND PATIENT CONSENT STATEMENT

This study was conducted in accordance with the guidelines of the UCSD Institutional Review Board (Study of Personalized Cancer Therapy to Determine Response and Toxicity, UCSD_PREDICT, NCT02478931) and any investigational interventions/therapies for which all patients gave written informed consent. Protocols were approved by the UCSD Internal Review Board.

## Supporting information


**Supporting information S1.** Supplementary material

## Data Availability

The data that support the findings of this study are available from the corresponding author upon reasonable request.
