# Supplementary material for: LAG‐3 transcriptomic expression patterns across malignancies: Implications for precision immunotherapeutics
Source: Cancer Med. 2023 May 3;12(12):13155–66. doi: 10.1002/cam4.6000 (PMC10315766; doi:10.1002/cam4.6000)
Supplement: Supplementary file 1 — Supporting information S1. Supplementary material [file CAM4-12-13155-s001.docx]

**Supplemental Table 1. Patient Characteristics**

| **All cancers** | N = 514 |
| --- | --- |
| Median Age (range) years | 61 (24-93) years |
| Men | 40% (N = 203) |
|  |  |
| **Tumor Histology** | **Number of patients (%)** |
| Breast cancer | 49 (10%) |
| Colorectal cancer | 140 (27%) |
| Lung cancer | 20 (4%) |
| Neuroendocrine cancer | 15 (3%) |
| Ovarian cancer | 43 (8%) |
| Pancreatic cancer | 55 (11%) |
| Sarcoma | 24 (5%) |
| Stomach cancer | 25 (5%) |
| Uterine cancer | 24 (5%) |
| All others (<10 samples per histology) | 120 (23%) |
